# Enhanced Blood Plasma Extraction Utilising Viscoelastic Effects in a Serpentine Microchannel

**DOI:** 10.3390/bios12020120

**Published:** 2022-02-14

**Authors:** Yuchen Dai, Haotian Cha, Michael J. Simmonds, Hedieh Fallahi, Hongjie An, Hang T. Ta, Nam-Trung Nguyen, Jun Zhang, Antony P. McNamee

**Affiliations:** 1 Queensland Micro-Nanotechnology Centre, Griffith University, Nathan, QLD 4111, Australia; y.dai@griffith.edu.au (Y.D.); haotian.cha@griffithuni.edu.au (H.C.); hedieh.fallahi@griffithuni.edu.au (H.F.); hongjie.an@griffith.edu.au (H.A.); nam-trung.nguyen@griffith.edu.au (N.-T.N.); 2 Biorheology Research Laboratory, Menzies Health Institute Queensland, Griffith University, Gold Coast, QLD 4222, Australia; m.simmonds@griffith.edu.au; 3 School of Environment and Science, Griffith University, Nathan, QLD 4111, Australia; h.ta@griffith.edu.au

**Keywords:** blood plasma separation, microfluidics, viscoelasticity, particle migration

## Abstract

Plasma extraction from blood is essential for diagnosis of many diseases. The critical process of plasma extraction requires removal of blood cells from whole blood. Fluid viscoelasticity promotes cell migration towards the central axis of flow due to differences in normal stress and physical properties of cells. We investigated the effects of altering fluid viscoelasticity on blood plasma extraction in a serpentine microchannel. Poly (ethylene oxide) (PEO) was dissolved into blood to increase its viscoelasticity. The influences of PEO concentration, blood dilution, and flow rate on the performance of cell focusing were examined. We found that focusing performance can be significantly enhanced by adding PEO into blood. The optimal PEO concentration ranged from 100 to 200 ppm with respect to effective blood cell focusing. An optimal flow rate from 1 to 15 µL/min was determined, at least for our experimental setup. Given less than 1% haemolysis was detected at the outlets in all experimental combinations, the proposed microfluidic methodology appears suitable for applications sensitive to haemocompatibility.

## 1. Introduction

Blood is a two-phase suspension of red blood cells (RBCs), white blood cells (WBCs), and platelets suspended in plasma. Given the high protein content, including serum albumin, globulins, and fibrinogen, within plasma, this fluid is often separated from whole blood and used for analytical purposes, including in vitro diagnostics. Blood plasma also contains glucose, electrolytes, hormones, carbon dioxide, and oxygen, so it is crucial for the intravascular osmotic effect which maintains the balance of electrolyte concentration and protects the body from infection and other blood diseases. However, the presence of cellular components inhibits diagnostic sensitivity, accuracy, and reproducibility. Hence, it is of great importance to remove blood cells from the whole blood and extract plasma for medical applications. Two conventional methods, namely centrifugal separation and membrane filtration, have become standard practice over the last century. Although the centrifugal technique is widely used in biological labs, such a device is bulky and involves a batch-based separation technique, thus it is limited for integration with other units to form a fully automated system for point of care diagnosis. Moreover, inappropriate centrifugation, despite the low likelihood, may cause haemolysis of RBCs, leading to contamination risks and misdiagnosis due to handling errors [1]. On the other hand, membrane filtration technique has finer structural details, but clogging and fouling issues inherent in this mode severely deteriorate filtration performance, especially for samples with high cellular content [2].

Microfluidic techniques refer to the systems that process fluids in channels with typical dimensions (width and height) in the order of 10 to 100 µm. The corresponding characteristic length scale enables precise manipulation of fluid and particles, such as cellular components of blood, and thus facilitates the purity and efficiency requirements for plasma separation. Microfluidic technology has consequently become a promising candidate for plasma separation in recent years [3,4,5]. 

Based on the origin of manipulating forces, microfluidics can be classified as either active or passive techniques. The former utilises external active forces, such as dielectrophoresis [6], magnetophoresis [7], or acoustophoresis [8], while passive methods exploit the intrinsic fluid dynamics of a sample [9,10]. Of particular relevance for passive microfluidic techniques are inertial microfluidics, which permit simple fabrication, low cost of development, and high throughput [11,12]. Note that within passive systems, the suspending medium is commonly a Newtonian fluid with a constant viscosity.

More recently, the migration of particles immersed in viscoelastic fluids has attracted increased attention due to the simple focusing equilibrium position caused by the intrinsic nonlinear elastic properties of viscoelastic fluids [13,14,15,16,17]. Leshansky et al. [18] observed that, when particles were suspended in viscoelastic fluids, flow within straight circular channels with negligible inertial conditions led to particle migration towards the centreline by the normal stress difference between the axis and the wall. In contrast, Yang et al. [19] reported that flow within straight rectangular channels of a viscoelastic suspension led to particles with different sizes migrating towards the corners, in addition to the centreline. The latter finding is more practical since microfluidic channels are mainly made in planar geometries, i.e., rectangular cross sections, due to standard photolithography and soft lithography. Based on the size-dependent migration in rectangular channels, viscoelastic fluids have been previously used to separate platelets from diluted whole blood [20], *E. coli* bacteria from RBCs [21], malaria parasites from WBCs [22], Jurkat cells from yeast cells [23], and Chlorella microalgae from *B. subtilis* bacteria [24]. In addition, Lu et al. [25,26] experimentally found that particles can be sorted by their shape/morphology in viscoelastic microfluidics [27,28,29]. Later, based on the simple shape-dependent migration principle, viscoelastic fluids were adopted to separate abnormal-shaped yeast cells from those that were regular shaped [30], incubated *Candida albicans* with germ tube formations from spherical candida cells [31], and cyanobacterial Anabaena with different rod aspect ratios [32]. Moreover, Yang et al. [33] demonstrated that elasto-inertial particles can also be sorted by their deformability, successfully isolating rigidified RBCs from fresh RBCs.

In contrast to sorting different cells into different locations, it is of more interest to focus all the cells into a single equilibrium position in terms of blood plasma separation. In Yang et al. [19], it was experimentally demonstrated that the number of equilibrium positions can be reduced to one (centreline) even in rectangular channels by increasing the fluid flow inertial force without altering any other condition. Additionally, secondary flows created in curved channels or straight channels with disturbance obstacles were found to facilitate migration and modify the equilibrium positions depending on the balance between the inertial lift and Dean drag forces [34,35]. Yuan et al. [36] then combined both Dean-flow and elasto-inertial effects, and successfully demonstrated the separation of blood plasma in a straight channel with asymmetrical expansion–contraction cavity arrays (ECCA channel); the purity of separated plasma was as high as 99.99%. However, the blood was highly diluted (~20 times) and the effect of blood dilution on plasma separation performance was not studied. Moreover, the haemocompatibility of the added viscoelasticity on blood processing (e.g., the intactness of RBCs) remains unknown.

In this work, we systematically investigated viscoelastic blood plasma extraction in a serpentine microchannel. Whole blood was diluted using a viscoelastic phosphate-buffered saline (PBS) solution dissolved with poly (ethylene oxide) (PEO). The influences of PEO solution concentration, blood dilution, and the inertial force on the blood plasma separation performance were investigated. Finally, the haemocompatibility of viscoelastic microfluidic blood plasma separation was examined by measuring levels of free haemoglobin within the separated plasma.

## 2. Theoretical Background

Among the theories of particle migration in Newtonian fluids, Asmolov’s solution [37] has been widely used to determine the lateral forces ($F_{L}$) exerted on particles immersed within Newtonian fluids in inertial flow [38], as follows:

$$F_{L}=\frac{\rho_{f}U_{m}^{2}a^{4}}{D_{h}^{2}}f_{L}\left(Re_{c},x_{c}\right)$$

where *ρ*_f_, *U*_m_, *a*, *D*_h_ denote the fluid density, mean velocity, spherical diameter of the particles, and hydraulic diameter of the channel, respectively. The lift coefficient *f*_L_(*Re*_c_, *x*_c_) is a function of the particle position within the cross section *x*_c_ and the channel Reynolds number *Re*_c_, which is expressed as:

$$Re_{c}=\frac{\rho_{f}U_{m}D_{h}}{\mu_{f}}$$

where *µ*_f_ represents the dynamic viscosity of the fluid.

In the presence of non-Newtonian viscoelastic fluids, an extra elastic force is exerted on particles. The elasticity can be characterised by Weissenberg number (Wi) in rectangular channel flows as [39]:

$$Wi=\lambda \dot{\gamma_{c}}=\frac{2\lambda Q}{hw^{2}}$$

where *λ*, $\dot{\gamma_{c}}$, *Q* denote the relaxation time of the fluid, characteristic shear rate and volumetric flow rate, respectively. For elasto-inertial flows in rectangular channels, both the first (*N*_1_ = *τ_xx_ −* *τ_yy_*) and second (*N*_2_ = *τ_yy_ −* *τ_zz_*) normal stress differences contribute to the particle migration. *τ_xx_*, *τ_yy_*, and *τ_zz_* denote normal stresses along the main flow, the velocity gradient, and vorticity direction, respectively. It is known that the second normal stress difference *N*_2_ leads to the development of the secondary motions in viscoelastic flows and it becomes negligibly small compared with *N*_1_ in diluted PEO solutions [40,41]. Thus, the lateral force *F*_E_ exerted on a particle by the normal stress difference is expressed as [42]

$$F_{E}\propto a^{3}\frac{\partial N_{1}}{\partial x}$$

## 3. Materials and Methods

### 3.1. Design and Fabrication of Microfluidic Devices

**Figure 1:**
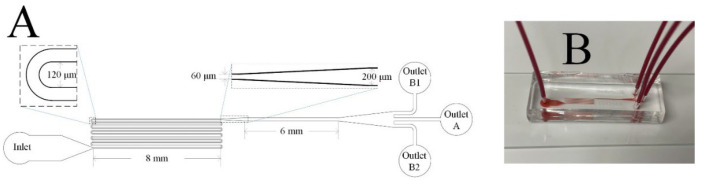
The microfluidic channel used in this study: (**A**) a schematic diagram; (**B**) an image of the fabricated microfluidic device during use with whole blood.

The microfluidic channel used in this study is illustrated in Figure 1. The channel consisted of a 90 mm serpentine section and a 6 mm straight section with a uniform depth of 70 µm. The width of each U-turn in the serpentine section was 120 µm. The width of the serpentine and straight channel were 60 µm and 200 µm, respectively. The middle outlet is marked as Outlet A, while the two side outlets are labelled as Outlet B1 and B2. All devices were fabricated by standard photolithography and soft lithography techniques [43], including silicon master fabrication, and poly-dimethylsiloxane (PDMS) replica molding as well as bonding through plasma oxidation.

### 3.2. Preparation of Viscoelastic Fluids

A 0.5% *w*/*v* PEO (average Mw~2 million Da, Sigma–Aldrich, St. Louis, MO, USA) in phosphate-buffered saline (PBS) solution was prepared by rapid mixing to form a uniform PEO solution with a concentration of 5000 parts per million (ppm). PEO was chosen as the viscoelastic agent because it is reported to be biocompatible [44] and is widely used in viscoelastic microfluidics [13]. The 5000 ppm PEO solution was subsequently mixed with whole blood and PBS at different ratios to achieve the following: blood dilutions (1/2, 1/3, 1/5, and 1/10); and PEO concentrations (100, 200, 500, 1000, and 2000 ppm). The viscosity of the varied blood–PEO suspensions was measured through a Brookfield DV3T rheometer with a CPA-40Z spindle. The results were assessed across a range of shear rates (75~1500 s^−1^) and is presented in the Supplementary Figure S1.

### 3.3. Preparation of Blood Cells

Blood was collected from healthy humans via venepuncture of a prominent vein in the antecubital region. Blood was collected into vacutainers containing 1.8 mg/mL of the anticoagulant K_2_EDTA. Experimental procedures were completed within 6 h of initial blood collection. All protocols were reviewed and approved by the Griffith University Human Research Ethics Committee (protocol number: 2021/773), which conforms with the Declaration of Helsinki.

### 3.4. Flow Cytometry and Haemoglobin Analysis

Cell counts and filtration efficiency was determined for samples collected from outlets A and B using flow cytometry and spectrophotometric haemoglobin analysis for each concentration of PEO. Flow cytometry was performed using a FC500 flow cytometer (Beckman Coulter, CA, USA), with RBCs gates identified using initial antibody staining of glycophorin A (CD35a) conjugated to fluorescein isothiocyanate. To obtain accurate cell counts, absolute cell counting beads (123count eBeads, Invitrogen, Waltham, MA, USA) were added to each sample prior to analysis. Absolute total events were used for analysis to compare cell counts between outlet A (centre) and outlet B (side) to gain a measure of filtration efficiency. 

To ensure the microfluidic device did not damage RBCs in the process of cell focusing and phase separation, samples collected from all outlets were assessed for haemolysis using the Harboe spectrophotometric method (PMID: 13646603) [45]. Briefly, the supernatant was isolated via centrifugation at 3000× *g* for 10 min, before being diluted 1:10 in 0.01% Na_2_CO_3_ solution. Each sample was thoroughly mixed and subsequently loaded into a microplate reader (FLUOstar Omega, BMG Labtech, Mornington, VIC, Australia) and absorbance was recorded at 380, 415, and 450 nm. Free haemoglobin concentration (mg/dL) was then calculated using the following formula: [Hb] = (167.2A_415_ − 83.6A_450_ − 83.6A_380_). Haemolysis was determined for central (A) and side (B) outlets relative to each respective total haemoglobin concentration.

### 3.5. Experimental Setup and Data Analysis

Blood samples diluted in PEO PBS were infused into the microfluidic device at specific flow rates by a syringe pump (neMESYS, Centoni GmbH, Korbußen, Germany). To avoid the presence of air bubbles inside the microchannel, which could adversely interrupt the flow characteristics, the microchannel as well as the connected tubing were fully filled with PBS before each experiment. In experiments, to reduce sedimentation of blood cells (which drastically influences the blood concentration within the microchannel), the syringe pump was regularly arranged from vertical to horizontal orientation, and vice versa. However, a mixing device should be integrated inline in practical applications, such as placing a magnetic stirrer bar in the sample chamber and activating it by a rotating magnetic field. The outputs from Outlet A and Outlet B were collected into two 2 mL tubes for each case. An inverted microscope (Nikon Eclipse Ti) equipped with a high-speed camera (Phantom Miro3, Vision Research) at an ultra-short (~20 µs) exposure time was utilised to monitor and record the flow of samples inside the microchannels. The open-source software ImageJ (National Institutes of Health, Bethesda, MD, USA) was adopted to analyse the captured videos. The images of each video were stacked so that the statistical distribution of blood cells could be visualised. The polynomial curve fitting method was adopted to fit the data of focusing performance through MATLAB.

## 4. Results and Discussions

### 4.1. Blood Plasma Extraction Mechanism

When blood samples diluted in PEO PBS solution flow through the serpentine channel, three forces exert on the cellular components, including an inertial lift force induced by the flow inertia [37], an elastic force caused by the viscoelastic fluid [18], and a Dean drag force resulting from the curved structure at each turn [34]. The Dean drag force is absent in the straight regions of each channel. In general, the inertial lift force focuses the particles at a certain distance away from the channel walls. Meanwhile, the viscoelastic force causes a lateral force on the blood cells by the first normal stress difference, leading to cell movement away from the wall. In addition, the serpentine channel contains curved regions with two opposite directions in a periodic pattern, resulting in the alternate directions of corresponding Dean drag forces. This process aids the focusing of cells towards the centreline of flow. Hence, under the synergistic effect of the three forces, the cellular components of the diluted blood are expected to be focused on the central area of the channel after a certain flowing distance. Finally, the blood plasma can be extracted from cell-free regions nearby two side walls and collected at the side outlets B1 and B2.

### 4.2. Fluid Viscoelastic Enhanced Focusing of Blood Cells

**Figure 2:**
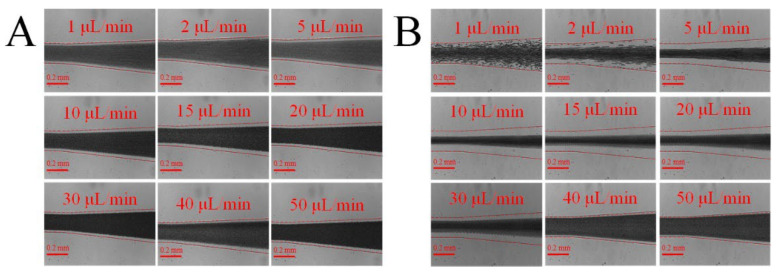
Blood cell distribution at the end of the serpentine channel at various flow rates: (**A**) whole blood diluted (×1/10) in PBS; (**B**) blood diluted (×1/10) in 500 ppm PEO PBS solution. The added viscoelasticity from PEO can facilitate the focusing of blood cells at channel centre, and the expanded cell-free area is beneficial for blood plasma extraction.

In this section, we studied whether the added fluid viscoelasticity facilitated the focusing of blood cells. The blood sample diluted in only PBS was selected to be the control, which is labelled as *C*_PEO_ = 0 ppm. We first compare it with whole blood diluted in 500 ppm PEO PBS solution. Figure 2 presents the distribution of blood cells after the serpentine region of the channel for the two cases, at the same blood concentration (1/10) and volume flow rates ranging 1 to 50 µL/min. The side walls of the microfluidic channel are identified in each test with a dashed line overlay. As observed, the added viscoelasticity from PEO facilitated the focusing of blood cells. For blood sample of *C*_PEO_ = 0 ppm, even with 10 times dilution, the cell-free layers are very narrow. However, the cell-free area is increased by the viscoelastic effect at certain range of flow rates for blood samples of *C*_PEO_ = 500 ppm. Furthermore, the total width of free cell layers first increases and then decreases with the increase of flow rate (Figure 2B), which indicates that the enhancement of cells focusing by viscoelasticity is also sensitive to flow rate.

**Figure 3:**
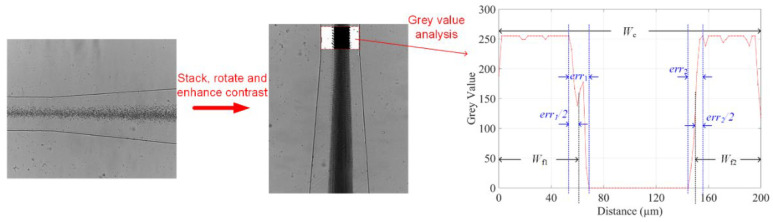
Image process and averaged greyscale value analysis to determine the boundaries of the cell-free areas. 50 frames of images captured by a high-speed camera were stacked. The greyscale values along the channel lateral position were obtained at the end of serpentine channel. The boundaries of the cell-free layers on two sides were determined by half of the error area width.

To quantify the focusing performance, a cell-free area ratio *δ* is introduced as follows:

$$\delta =\frac{W_{f}}{W_{c}}=\frac{W_{f1}+W_{f2}}{W_{c}}$$

where *W*_f_ denotes the total averaged cell-free layer width, which is the sum of the two cell-free layer widths *W*_f1_ and *W*_f2_ nearby channel walls. *W*_c_ represents the channel width. To calculate *W*_f_, we first stacked each frame of the micrographs into a single figure. A rectangular region of interest was then selected at a discrete point before the expansion. Finally, the profile of grey values along the channel’s width was obtained to determine the cell-free area with the error areas considered. Figure 3 illustrates the process using the data from the 1/10 blood concentration and 500 ppm PEO at flow rate of 10 µL/min as an example. Since the greyscale value of 0 represents black, it is not difficult to determine the widths of the error areas (*err_1_* and *err_2_*), and then the total width of cell-free layers on two sides *W*_f_ can be estimated, so as *δ*.

**Figure 4:**
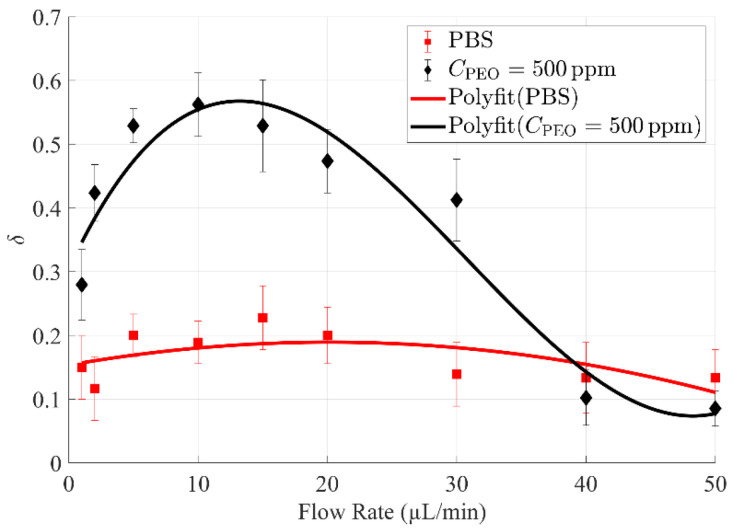
Comparison of cell-free area ratio *δ* of diluted (×1/10) whole blood samples in PBS and 500 ppm PEO PBS, respectively. *δ* of diluted blood in 500 ppm PEO PBS is significantly higher than that in only PBS at low to moderate flow rates (1~30 µL/min).

By repeating the procedures above, the effect of flow rate on the cell-free area ratio *δ* can be obtained (Figure 4). As presented, the cell-free area ratio *δ* of 10 times diluted blood in 500 ppm PEO PBS is significantly higher than that in only PBS at low to moderate flow rates (1~30 µL/min), while a narrowed cell-free area ratio *δ* was found at high flow rates (40 and 50 µL/min). Importantly, a peak value of *δ* was identified for the focusing performance of 10 times diluted blood in 500 ppm PEO PBS, as demonstrated by the fitting curve. Specifically, when the flow rate was 10 µL/min, *δ* of 10 times diluted blood in 500 ppm PEO PBS was at its highest value of approximately 0.56, which is more than two times of the peak *δ* value (around 0.23) of PBS.

### 4.3. Effects of PEO Concentration and Blood Dilution

**Figure 5:**
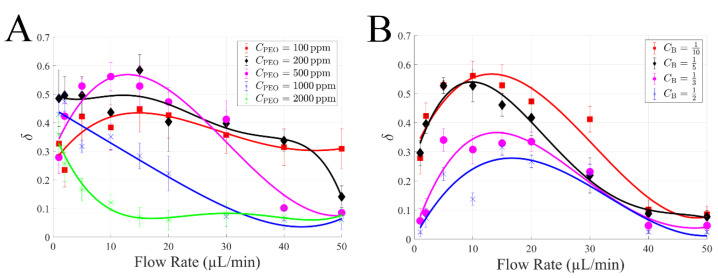
The effects of PEO concentration and blood dilution number on cell-free area ratio *δ*: (**A**) 10 times diluted blood with various PEO concentrations (100, 200, 500, 1000, and 2000 ppm); (**B**) various blood dilutions (1/2, 1/3, 1/5, and 1/10) at 500 ppm PEO concentration.

We further investigated the effects of PEO concentration and blood dilution on blood cells’ focusing performance. Various PEO concentrations and blood dilutions demonstrated a similarly peaked response (Figure 5); the cell-free area ratio *δ* generally shows an initial increase and subsequent decrease when flow rate increased, except for the case with the highest PEO concentration (*C*_PEO_ = 2000 ppm) which demonstrates a consistently decreasing trend. Additionally, low PEO concentrations (*C*_PEO_ = 100, 200, and 500 ppm) demonstrated superior performance compared to counterparts with high PEO concentration (*C*_PEO_ = 1000, 2000 ppm). Specifically, the highest *δ* was approximately 0.44 of *C*_PEO_ = 100 ppm at 15 µL/min, 0.58 of *C*_PEO_ = 200 ppm at 15 µL/min, and 0.56 of *C*_PEO_ = 500 ppm at 10 µL/min. In general, optimal *δ* were found within low to moderate flow rates, and this optimised flow rate decreased from 15 to 1 µL/min when *C*_PEO_ increased from 100 to 2000 ppm (Figure 5A). Furthermore, among the various blood dilution cases, the 10 times diluted blood consistently shows a superior effect to others, as presented in Figure 5B. This is not unexpected, since larger dilution has fewer cells, thus leading to larger cell-free layers. Similarly, optimal focusing performances were found within low to moderate flow rates ranging from 5 to 15 µL/min. Among all the cases, the highest cell-free area ratio *δ* was approximately 0.58 with 200 ppm PEO concentration and 10 times diluted blood at 15 µL/min.

### 4.4. Blood Plasma Extraction and Haemoglobin Analysis

**Figure 6:**
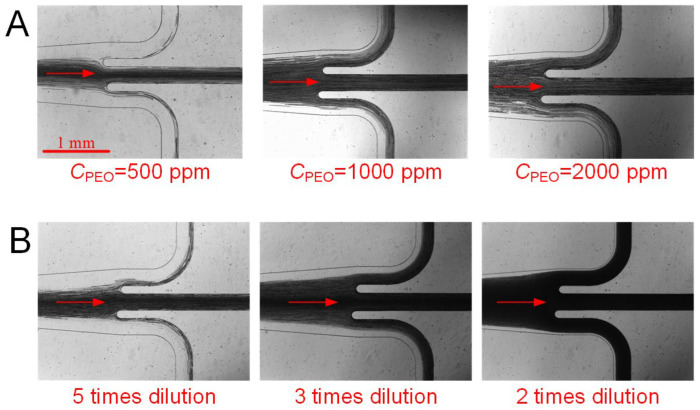
Focusing of blood cells and plasma extraction at the bifurcation outlets: (**A**) 10 times diluted blood with various PEO concentrations; (**B**) various blood dilutions with 200 ppm PEO concentration.

Figure 6 presents the focusing of blood cells and plasma separation at the bifurcation outlets at 15 µL/min for several typical cases. As observed, at a moderate flow rate, either increasing the PEO concentration or decreasing blood dilution results in wider cell layers. Consequently, the collection of cell-free plasma from blood becomes less effective. Indeed, in the present “best case scenario” (10 times dilution of blood in 500 ppm PEO), several blood cells may still be observed at the two-sided outlets, likely due to a suboptimal flow resistance ratio between the outlets; thus, opportunities that exist to optimise the outlets should improve performance of plasma separation.

**Figure 7:**
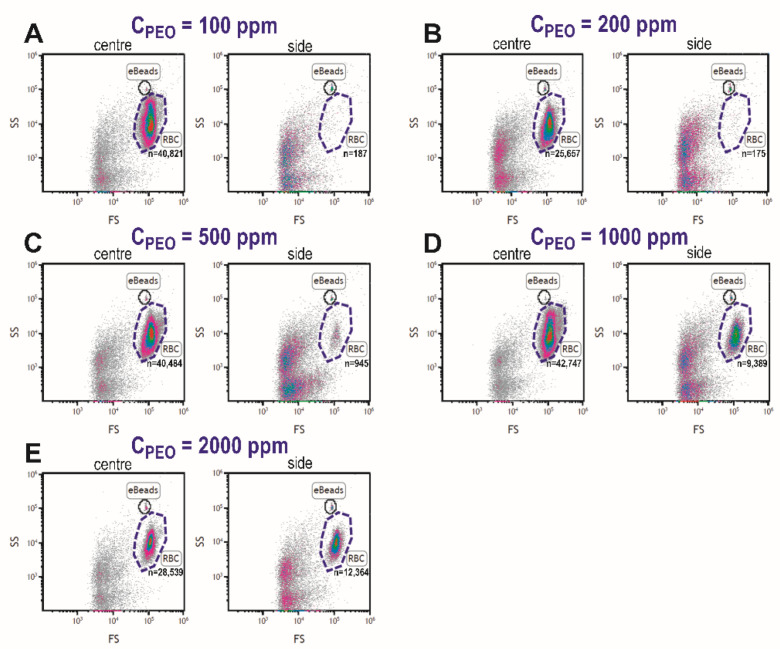
Flow cytometric cell counts in central and side outlets of the microfluidic device for blood samples suspended at 1:10 dilution of increasing concentrations of PEO; 100 (**A**), 200 (**B**), 500 (**C**), 1000 (**D**), and 2000 ppm (**E**). RBCs: red blood cells; FS: forward scatter; SS: side scatter.

After determining the optimal viscoelastic focusing at 15 µL/min, the blood plasma separation was conducted for diluted blood samples (1/10) with different PEO concentrations and the collected plasma from the different outlets was examined. Figure 7 displays the flow cytometric data for central and side outlets for increasing PEO concentration, where gated regions identify RBCs populations and eBeads. Absolute cell counts (*n*) in the RBCs region increased for samples collected from the side outlet for the blood sample with PEO concentration ≥500 ppm.

**Figure 8:**
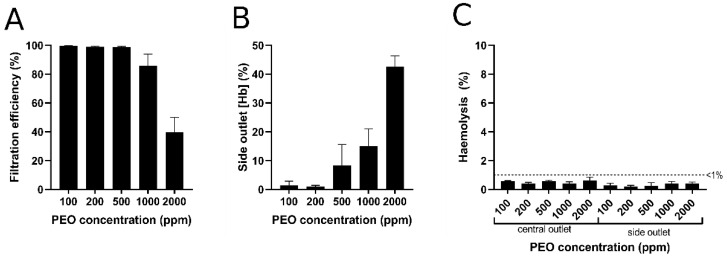
Filtration efficiency as determined by comparative RBCs counts (**A**) and relative total haemoglobin concentration (**B**) of the side outlet versus central outlet of the microfluidic device for each PEO concentration. (**C**) Measured cell-free haemoglobin (i.e., haemolysis) at the central and side outlets of the microfluidic device for each PEO concentration. (Hb): haemoglobin concentration. Error bars represent standard error of the mean (*n* = 3).

To determine an index of filtration efficiency for plasma extraction, absolute cell counts in the side outlet were presented relative to total cell counts in the central channel for each PEO concentration (Figure 8A). The filtration efficiency into the side channel decreased at PEO concentrations ≥500 ppm (Figure 8A), which can be supported by the fact that the total RBCs haemoglobin content in the side channel increased (Figure 8B). Although total measured haemoglobin content increased at PEO concentrations of 500, 1000, and 2000 ppm, no substantial haemolysis (i.e., cell-free haemoglobin; <1%) was detected in the outlet of any condition (Figure 8C), indicating that the microfluidic device does not rupture RBCs in the process of viscoelastic focusing and plasma extraction. Based on the data of filtration efficiency and haemolysis in Figure 8, the blood samples with PEO concentrations of 100 and 200 ppm are optimal for blood plasma extraction. 

While this study mainly focused on RBCs movement and blood plasma extraction for haemoglobin detection, movement of other blood cells (WBCs and platelets) must also be considered. Since particle size dictates cell centreline focusing or margination, WBCs (which are generally larger than RBCs) should focus towards the centreline, and platelets (which are smaller than RBCs) should have less alignment. Therefore, there are likely fewer WBCs and more platelets in the separated plasma, evidenced by the flow cytometric data in Figure 7. In our data collection, platelets were not qualitatively identified due to their size; however, the spectrophotometric analyses for haemoglobin detection are not sensitive to platelet concentration or the presence of WBCs [46].

The processing throughput of the proposed device is far less than standard centrifugation. However, many analytical methods only require small volumes of isolated plasma (less than 30 µL; e.g., the Harboe spectrophotometric method for haemoglobin assessment). While increasing the throughput of the device would not be required for future microfluidic devices with embedded inline analytics, one potential solution for increasing throughput would be to implement multiple parallel channels to amplify the output. The proposed plasma extraction microfluidic technology in the present study is part of a staged theme of works seeking to develop a fully-automated bedside point-of-care free-haemoglobin quantification system. Given the developments towards inline analytics, we believe that the relatively low throughput may not be a significant issue. The advantages of microfluidic plasma extraction technology and potential integration with other functional units and automation will likely increase clinical efficiency and reduce human intervention.

Finally, it is plausible that the current microfluidic device could be deployed to ascertain blood phase separation for cell focusing and plasma isolation, with prospects to integrate inline plasma analytics at side outlets for development into a future stand-alone device for blood analytics.

## 5. Conclusions

The current study investigated viscoelastic blood plasma extraction in a sheathless serpentine microchannel. The influences of PEO solution concentration, blood dilution, and flow rate on the cell focusing performance were tested. The experimental data demonstrated that the cell focusing performance, in general, shows a biphasic trend (first increasing and then decreasing) when flow rate increases, at least for the measured PEO concentrations and blood dilutions used. Hence, the optimal cell focusing performance for each case was determined with the flow rate ranging from 1 to 15 µL/min. Furthermore, the highest cell focusing performance among all the cases was found to be approximately 0.58 with 200 ppm PEO concentration and 10 times diluted blood at 15 µL/min flow rate. Finally, the haemocompatibility results showed that only <1% haemolysis was detected in the outlet region of any condition, indicating that the microfluidic device does not rupture RBCs in the process of viscoelastic focusing and plasma extraction.

## Data Availability

The data presented in this study are available on request from the corresponding authors.

## Supplementary Materials

The following supporting information can be downloaded at: https://www.mdpi.com/article/10.3390/bios12020120/s1, Figure S1: (A) Blood viscosity at different PEO concentrations. Blood dilution is 1/10. (B) Blood viscosity at different dilutions. PEO concentration is 500 ppm.
