# Supplementary material for: Enhanced Blood Plasma Extraction Utilising Viscoelastic Effects in a Serpentine Microchannel
Source: Biosensors (Basel). 2022 Feb 14;12(2):120. doi: 10.3390/bios12020120 (PMC8869685; doi:10.3390/bios12020120)
Supplement: Supplementary file 1 [file biosensors-12-00120-s001.zip › biosensors-1536644-supplementary.pdf]

biosensors

Article

Enhanced Blood Plasma Extraction Utilising Viscoelastic Effects in a Serpentine Microchannel

Yuchen Dai [1], Haotian Cha [1], Michael J. Simmonds [2], Hedieh Fallahi [1], Hongjie An [1], Hang T. Ta [3], Nam-Trung Nguyen [1], Jun Zhang [1,*] and Antony P. McNamee [2,*]

[1] Queensland Micro-Nanotechnology Centre, Griffith University, Nathan, QLD 4111, Australia; y.dai@griffith.edu.au (Y.D.); haotian.cha@griffithuni.edu.au (H.C.); hedieh.fallahi@griffithuni.edu.au (H.F.); hongjie.an@griffith.edu.au (H.A.); nam-trung.nguyen@griffith.edu.au (N.-T.N.)
[2] Biorheology Research Laboratory, Menzies Health Institute Queensland, Griffith University, Gold Coast, QLD 4222, Australia; m.simmonds@griffith.edu.au
[3] School of Environment and Science, Griffith University, Nathan, QLD 4111, Australia; h.ta@griffith.edu.au
* Correspondence: jun.zhang@griffith.edu.au (J.Z.); a.mcnamee@griffith.edu.au (A.P.M.)

Citation: Dai, Y.; Cha, H.; Simmonds, M.J.; Fallahi, H.; An, H.; Ta, H.T.; Nguyen, N.-T.; Zhang, J.; McNamee, A.P. Enhanced Blood Plasma Extraction Utilising Viscoelastic Effects in a Serpentine Microchannel. *Biosensors* **2022**, *12*, 120. https://doi.org/10.3390/bios12020120

Received: 22 December 2021
Accepted: 12 February 2022
Published: 14 February 2022

Publisher's Note: MDPI stays neutral with regard to jurisdictional claims in published maps and institutional affiliations.

Copyright: © 2022 by the authors. Submitted for possible open access publication under the terms and conditions of the Creative Commons Attribution (CC BY) license (https://creativecommons.org/licenses/by/4.0/).

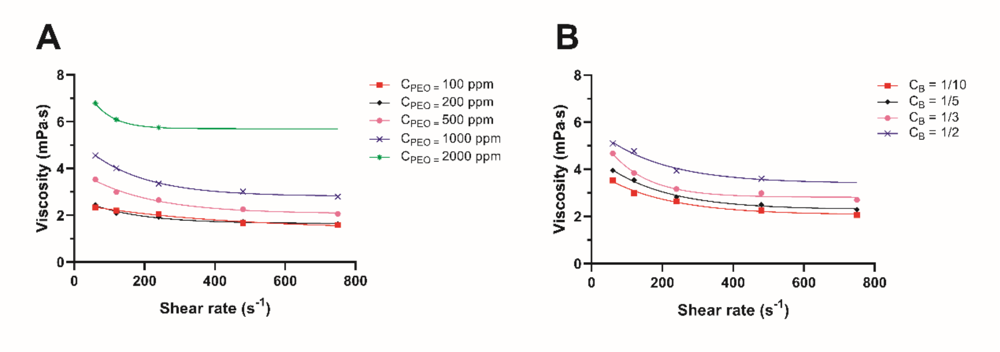

Figure S1

Figure S1. (A) Blood viscosity at different PEO concentrations. blood dilution is 1/10. (B) Blood viscosity at different dilutions. PEO concentration is 500 ppm.
